# *In situ* techniques reveal the true capabilities of SOFC cathode materials and their sudden degradation due to omnipresent sulfur trace impurities†

**DOI:** 10.1039/d2ta03335f

**Published:** 2022-06-23

**Authors:** Christoph Riedl, Matthäus Siebenhofer, Andreas Nenning, Alexander Schmid, Maximilian Weiss, Christoph Rameshan, Andreas Limbeck, Markus Kubicek, Alexander Karl Opitz, Juergen Fleig

**Affiliations:** Institute of Chemical Technologies and Analytics, TU Wien Getreidemarkt 9-E164 1060 Vienna Austria christoph.riedl@tuwien.ac.at; Institute of Materials Chemistry, TU Wien Getreidemarkt 9-E165-PC 1060 Vienna Austria; CEST Kompetenzzentrum für Elektrochemische Oberflächentechnologie GmbH, TFZ – Wiener Neustadt Viktor-Kaplan-Strasse 2 2700 Wiener Neustadt Austria

## Abstract

In this study, five different mixed conducting cathode materials were grown as dense thin films by pulsed laser deposition (PLD) and characterized *via in situ* impedance spectroscopy directly after growth inside the PLD chamber (i-PLD). This technique enables quantification of the oxygen reduction kinetics on pristine and contaminant-free mixed conducting surfaces. The measurements reveal excellent catalytic performance of all pristine materials with polarization resistances being up to two orders of magnitude lower than those previously reported in the literature. For instance, on dense La_0.6_Sr_0.4_CoO_3−*δ*_ thin films, an area specific surface resistance of ∼0.2 Ω cm^2^ at 600 °C in synthetic air was found, while values usually >1 Ω cm^2^ are measured in conventional *ex situ* measurement setups. While surfaces after i-PLD measurements were very clean, ambient pressure X-ray photoelectron spectroscopy (AP-XPS) measurements found that all samples measured in other setups were contaminated with sulfate adsorbates. *In situ* impedance spectroscopy during AP-XPS revealed that already trace amounts of sulfur present in high purity gases accumulate quickly on pristine surfaces and lead to strongly increased surface polarization resistances, even before the formation of a SrSO_4_ secondary phase. Accordingly, the inherent excellent catalytic properties of this important class of materials were often inaccessible so far. As a proof of concept, the fast kinetics observed on sulfate-free surfaces were also realized in *ex situ* measurements with a gas purification setup and further reduces the sulfur concentration in the high purity gas.

## Abstract 2

In this study, the effect of sulphur contamination on pristine SOFC cathode materials is revealed by *in situ* impedance spectroscopy directly after growth inside the PLD chamber (i-PLD) and ambient pressure XPS measurements (AP-XPS).

## Graphical abstract

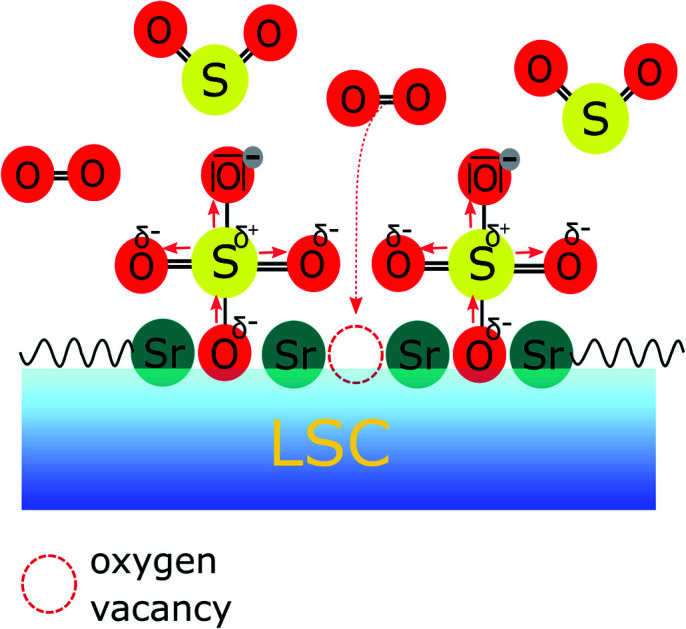

## 1 Introduction

The optimization of solid oxide fuel and electrolysis cells (SOFC/SOECs) is an important step towards efficient and sustainable energy conversion and storage.^1^ In the case of SOFCs, the main interest of research activities includes lowering their operation temperature to intermediate temperatures (500–600 °C). Lower operation temperatures reduce start-up times, allow the usage of materials which are cheaper to fabricate, and can improve the durability of cells.^2^ Nevertheless, this also limits the transport and reaction kinetics and thus the current output of the cell, as the relevant electrochemical processes are thermally activated. Typically, cathode processes have the highest activation energy and thus dominate the area specific resistance (ASR) of cells at lower temperature. Thus, highly active cathode materials are required, offering both low polarization resistances at intermediate temperatures and high long term stability of the resistance values.^2,3^ While currently La_1−*x*_Sr_*x*_MnO_3−*δ*_ (LSM) or La_1−*x*_Sr_*x*_Co_1−*y*_Fe_*y*_O_3−*δ*_ (LSCF) based composites are used as cathode materials in SOFCs,^4^ several recent studies have investigated the applicability of numerous alternatives. For example, perovskite-type mixed ionic electronic conducting (MIEC) materials such as SrTi_*x*_Fe_1−*x*_O_3−*δ*_ (STF),^5–7^ La_1−*x*_Sr_*x*_FeO_3−*δ*_ (LSF),^8–11^ and La_1−*x*_Sr_*x*_CoO_3−*δ*_ (LSC)^12–15^ or fluorite-type materials such as Pr_*x*_Ce_1−*x*_O_2−*δ*_ (PCO)^16–19^ are promising candidates for air electrodes.

Several studies have shown that LSC and STF can exhibit enormously fast oxygen reduction kinetics shortly after fabrication of thin film electrodes^5^ (*e.g.*: about 1 Ω cm^2^ for dense LSC thin films at 600 °C in air^14^). Moreover, *in situ* impedance measurements inside of the PLD chamber revealed even lower resistances (1 Ω cm^2^ at 600 °C and 0.04 mbar O_2_)^20^ However, such low resistances could never be reproduced in *ex situ* setups. Additionally a strong increase of their polarization resistance is observed over time, which is usually called “degradation”.^11,14,21,22^ Manifold reasons for degradation have been proposed; for example, studies have suggested Sr segregation to the surface and the concomitant formation of parasitic SrO phases to be a main reason for degradation.^21,23–28^ Moreover, it is well established that secondary phase formation due to sulfur species such as SO_2_ (ref. 11, 22 and 29–43) as well as silicon^18,44^ and chromium contamination^45,46^ can strongly decrease the performance of SOFC cathodes.

In most of the studies mentioned above, either thin film electrodes are deposited on single crystalline substrates by PLD, or porous samples are prepared by sintering and are then transferred to *ex situ* measurement setups. This approach suffers from several limitations as electrodes might already be exposed to contamination during sample transfer or heating and equilibration in the *ex situ* measurement setup, which may strongly alter the oxygen exchange kinetics and may even change oxygen reduction pathways. Different preparation conditions, but importantly, also different degradation states are almost certainly a main reason why polarization resistances obtained from different studies in various research groups are often afflicted by substantial scatter. This severely complicates a comparison of properties of different materials.

To avoid the above-mentioned obstacles, this study applies *in situ* impedance spectroscopy during pulsed laser deposition (i-PLD),^11–13^ a technique where thin film electrodes are characterized during or directly after growth in the chamber of the PLD.^11–13,47^ In contrast to *ex situ* experiments, this ensures pristine and thus extremely clean surfaces and enables an otherwise impossible observation of the inherent polarization resistance of electrode materials. LSC, LSF, Pt doped LSF, STF and PCO electrodes were studied under exactly the same conditions inside the PLD chamber, and all materials exhibit by far faster oxygen reduction kinetics (*i.e.* lower electrode polarization resistances) than those previously reported in *ex situ* studies. Moreover, LSC electrodes were investigated by combined electrochemical and ambient pressure X-ray photoelectron spectroscopy (AP-XPS) measurements, which allow to simultaneously study the surface chemistry of the electrodes by XPS and the polarization resistance of the electrodes by impedance measurements. This novel combination of methods unveils sulfur adsorbates due to omnipresent gas phase impurities as the reason for the strong initial degradation of pristine surfaces and advances the general understanding of the oxygen exchange kinetics and their degradation on SOFC cathode materials.

## 2 Experimental section

Powders were prepared following a synthesis method already described in the literature. LSF, LSC, Pt doped LSF and PCO were synthesized *via* a (modified) Pechini route,^8,18^ STF *via* solid state synthesis.^48^ All chemicals were purchased from Sigma-Aldrich with 99.995% purity or higher. The obtained powders were calcined at 800 °C in air, pressed into PLD targets by isostatic pressing (150 MPa) and sintered at 1200 °C in air for 12 h. The crystallinity of the deposited thin films was examined by X-ray diffraction analysis.

As electrolytes, yttria stabilized zirconia single crystals (YSZ (100), 0.5 × 0.5 × 0.05 cm^3^, 9.5 mol% Y_2_O_3_) (CrysTec, Germany) were used. Before PLD thin film growth, platinum grids (15|5 μm or 25|10 μm mesh|strip width) were prepared on the single crystals by lift-off photo lithography and magnetron sputtering of 5 nm titanium and 100 nm platinum (BAL-TEC MED 020, Liechtenstein). As a counter electrode, nano-porous La_0.6_Sr_0.4_CoO_3−*δ*_ (LSC) was deposited on the bottom side of the crystals by PLD with a KrF excimer laser (Complex Pro 201F, wavelength 248 nm) at 450 °C (substrate temperature measured with a pyrometer) and 0.4 mbar O_2_ with a target to substrate distance of 5 cm.^12^ At the beginning of every experiment, a 40 nm thick LSC layer was deposited onto the substrate (mixed ionic/electronic conductor with >1000 S cm^−1^ electronic conductivity) to ensure sufficient in-plane conduction for the subsequent working electrode layer. The working electrodes (*i.e.* La_0.6_Sr_0.4_CoO_3−*δ*_ (LSC), La_0.6_Sr_0.4_FeO_3−*δ*_ (LSF), Pt doped LSF (La_0.6_Sr_0.4_Fe_0.985_Pt_0.015_O_3−*δ*_), SrTi_0.3_Fe_0.7_O_3−*δ*_ (STF) and Pr_0.1_Ce_0.9_O_2−*δ*_ (PCO)) were then deposited on top of the LSC layer by laser ablation (2 Hz) at 600 °C substrate temperature, a target to substrate distance of 6 cm and 0.04 mbar O_2_. During the working electrode growth, the substrate temperature was monitored *via* the high frequency intercept of the measured impedance spectra using the known temperature dependence of the ionic conductivity of YSZ.^12,49^ The growth rate was determined *ex situ* on samples specially prepared for this purpose by measurements with a profilometer (DekTakXT, Bruker, USA) after deposition of a certain number of pulses: LSF 1 nm/71 pulses; LSC 1 nm/33 pulses; STF 1 nm/60 pulses; Pt doped LSF 1 nm/77 pulses and PCO 1 nm/ 18 pulses.

Impedance measurements were conducted with an Alpha-A high performance frequency analyser in combination with an electrochemical test station POT/GAL 30 V/2 A (both: Novocontrol Technologies, Germany). For i-PLD measurements (*in situ* impedance spectroscopy during PLD), a sample was placed on a PLD heater and covered with an alumina mask (cut-out 0.45 × 0.45 cm^2^) to prevent short circuiting around the edges of the sample during film deposition.^47^ The working electrode was contacted with a PtIr-needle, and the counter electrode was placed on the heating stage, which was brushed with platinum paste. Impedance spectra were recorded in the frequency range from 10^6^ to 10^−1^ Hz (10^−2^ Hz, if needed) with an AC amplitude of 10 mV root mean square and a resolution of 5 or 10 points per frequency decade.

For *ex situ* measurements, electrodes fabricated with the same parameters as the i-PLD electrodes were clamped between platinum meshes in a measurement setup made of quartz. The temperature was determined from the high frequency intercept in the impedance spectra representing mainly the YSZ single crystal resistance^12,49^ and double checked with a type S thermocouple mounted at a <1 cm distance to the sample.

Gas cylinders (50 L; 200 bar) filled with synthetic air (5.0) and oxygen (5.0) (Messer Austria GmbH) and equipped with standard pressure regulators were used as feed gas supply in all experiments. For further purification of the feed gas, a self-made gas cleaning equipment was constructed. The gas was first bubbled through three gas washing bottles containing saturated calcium hydroxide solution, saturated copper sulfate solution and bi-distilled water, respectively. The gas was then piped through a cooling trap filled with frozen ethanol (−120 °C) to freeze out water vapour. The gas was further cleaned with a solid-state adsorption column containing milled calcined LSF powder and subsequently dried in a high-capacity moisture trap (Restek™). It was found that all parts of the setup contribute to the removal of sulfur from the measurement gas, with the washing bottles being the most important part of the setup.

To measure the sulfur compound content of the used gases, the feed gas was bubbled (54 sccm) through two gas washing bottles equipped with a frit (porosity 3) containing 150 mL of distilled water with 3% H_2_O_2_, which allowed to getter sulfur compounds from the feed gas as SO_4_^2−^. The concentration of SO_4_^2−^ in the gas washing bottles was then measured *via* inductively coupled plasma mass spectrometry (ICP-MS). These measurements were carried out on an iCAP Q quadrupole ICP-MS (Thermo Fischer Scientific, Germany), coupled with an autosampler (SC-2-DX, ESI, USA) combined with an ESI Fast valve (ESI, USA), and the data were collected using the instrument software, Qtegra Version 2.8. The samples were diluted with 1% (v/v) HNO_3_, and 1 ng g^−1^ indium was added as the internal standard. For sulfur, the ^32^S^16^O^+^ and ^34^S^16^O^+^ ions were measured in the reaction mode by introducing a mixture of 10% oxygen in helium into the collision/reaction cell of the instrument. The sulfur concentration was quantified using standard addition. The detected amount of sulfur in the first gas washing bottle was 20 times higher than in the second gas washing bottle, thus we could safely assume that indeed virtually the entire amount of SO_2_ in the gas stream was captured in both washing bottles.

*In situ* XPS spectra were acquired in a lab-based AP-XPS setup with a PHOIBOS NAP photoelectron analyser (SPECS, Germany) and a monochromated Al K-alpha XR 50 MF (microfocus) X-ray source. Therein, the solid oxide cell was mounted on a special sample holder with a centred 4.9 × 4.9 mm^2^ sized hole for heating with a near-infrared diode laser.^50^ Electrical contacts for working and counter electrodes were established using Pt–Ir and sintered platinum paste tips, respectively. The temperature of the sample was controlled *via* the conductivity of the YSZ electrolyte. Although, in principle, the setup can handle up to 20 mbar gas phase pressure, XPS spectra were measured from 5 × 10^−6^ to 5 × 10^−3^ mbar O_2_, in order to minimize the concentrations of any contaminant species (especially SO_2_). XPS spectra were collected at an analyzer pass energy of 30 eV, which provided a reasonable balance of the count rate and energy resolution.

## 3 Results

### 3.1 Surface-related polarization resistance of pristine electrodes compared by i-PLD impedance measurements

**Fig. 1:**
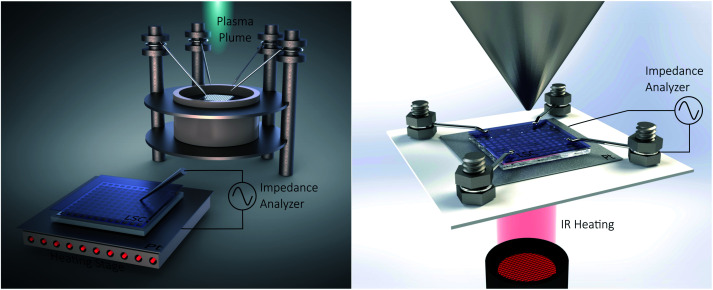
Sketch of the i-PLD setup (l.h.s) and the AP-XPS setup (r.h.s) with simultaneous electrochemical characterization.

**Fig. 2:**
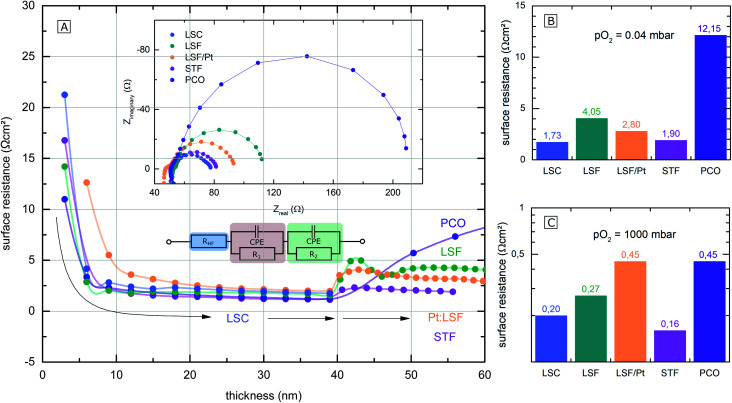
(A) Surface-related polarization resistance of the investigated electrodes, determined by i-PLD measurements, plotted over the electrode thickness. At first, 40 nm of LSC were deposited, followed by different other electrode materials (LSF (green), Pt doped LSF (orange), STF (pink) and PCO (purple)). (B) and (C) Bar charts of calculated area specific surface resistances of the electrodes at 0.04 mbar and 1000 mbar O_2_.

The polarization resistances of different electrodes La_0.6_Sr_0.4_CoO_3−*δ*_ (LSC), La_0.6_Sr_0.4_FeO_3−*δ*_ (LSF), Pt doped LSF (La_0.6_Sr_0.4_Fe_0.985_Pt_0.015_O_3−*δ*_), SrTi_0.3_Fe_0.7_O_3−*δ*_ (STF) and Pr_0.1_Ce_0.9_O_2−*δ*_ (PCO) were measured directly inside the vacuum chamber of the PLD setup at 600 °C and 0.04 mbar O_2_ or 1000 mbar O_2_. The corresponding i-PLD setup is depicted in Fig. 1 (l.h.s); details on mounting and contacting the samples are described elsewhere.^13,50^ During i-PLD measurements, electrodes are grown stepwise, and their polarization resistance is simultaneously monitored by *in situ* impedance measurements under deposition conditions. i-PLD measurements allow us to compare different thin film electrodes in their truly pristine state, which means unaltered by degradation and virtually free of any surface contamination. For all samples, first, a 40 nm thick LSC base layer was deposited to guarantee sufficient electronic in-plane conductivity and comparable surface morphologies. Subsequently, the electrode material of interest was deposited on top of this thin layer. Fig. 2 displays the surface-related polarization resistance resulting from the impedance measurements accompanying the deposition of the LSC base layer and the material of interest. A rather constant resistance is found for the base layer after *ca.* 20 nm, but significant changes of the surface properties were observed right after changing the material. Non monotonous changes of the polarisation resistance (*e.g.* for LSF) might be due to strain and growth effects but were not further investigated in this study. The deposition was continued till a material-specific critical thickness was reached, where saturation was observed.

The surface-related polarization resistances were determined from the recorded impedance spectra. These show very similar features for all materials that could be described with the same equivalent circuit; see the inset of Fig. 2(A). Details on the interpretation of the impedance of i-PLD studies can be found elsewhere. Essentially, all spectra exhibit a high frequency intercept of about 50 Ω assigned to the ionic conductivity of the YSZ single crystal used as a substrate plus the ohmic resistance of the electronic current collection. The dominant pronounced low frequency semicircle was fitted to a single *R*|CPE element (CPE = constant phase element). As has been shown in several studies,^5,10,51^ this resistance is inversely proportional to the oxygen exchange reaction rate on the surface of the electrode. In the following, only this resistance is considered and termed surface resistance or surface-related polarization resistance.

The parallel chemical capacitance describes the capability of the MIEC material to alter its oxygen stoichiometry upon changes of the oxygen chemical potential (*e.g.* by the AC signal during impedance spectroscopy).^9^ In the medium frequency range, a more or less pronounced shoulder was observed, which is most likely associated with the ion transfer across the electrode/electrolyte interface and which was described by an additional *R*|CPE element. For calculation of the area-specific resistance, the measured surface resistance was normalized to the area of the thin film, which was deposited directly on the YSZ (25–50% of the total electrode area). This way of normalization has already been proven meaningful in previous studies, where it was demonstrated that only the area above the YSZ is electrochemically active for oxygen reduction, while the regions above the current collector grid are inactive due to relatively low ionic in-plane conduction.^8^

The surface-related polarization resistances of all materials at 0.04 mbar O_2_ (*i.e.* at deposition pressure) and at 1000 mbar O_2_ (measured after deposition but in the PLD chamber) are plotted in the bar charts in Fig. 2(B) and (C) for a temperature of 600 °C. While at 0.04 mbar O_2_, the perovskite electrodes (LSF, LSC, STF and Pt doped LSF) show significantly lower surface-related polarization resistances than PCO, at 1000 mbar, the differences in the surface resistance mostly vanish. The different *p*(O_2_) dependence of this resistance for PCO can be attributed to the very different defect chemistry of PCO^16^ compared to the perovskite-type electrodes.^9^ An in-depth mechanistic discussion of the relation between polarization resistance and defect chemistry is beyond the scope of the present work but can be found in a separate study.^20^

### 3.2 Comparison of i-PLD surface resistances with literature data

**Fig. 3:**
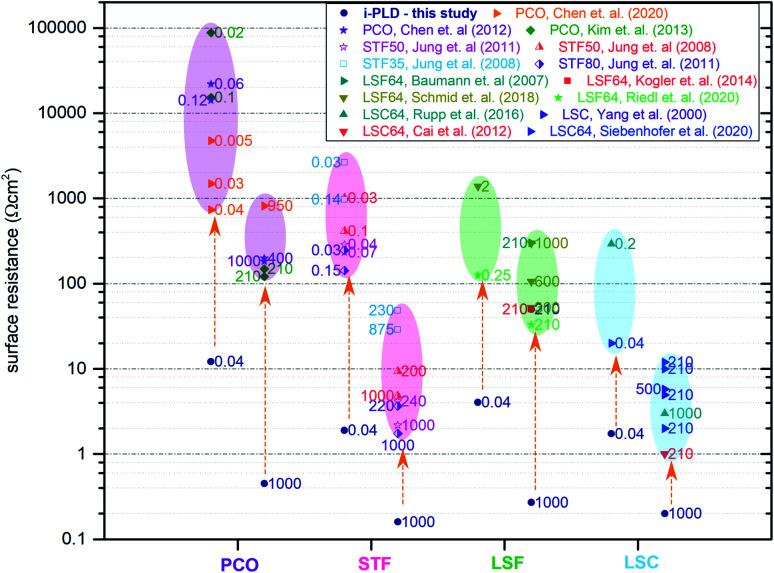
Electrode surface resistances from the present study (i-PLD) at 0.04 mbar and 1000 mbar O_2_ compared with values from figures published in the literature: PCO,^17,52,53^ STF,^5,6^ LSF,^8,51,54^ and LSC.^13,14,55,56^ Values from the literature are all measured in standard *ex situ* measurement setups. Numbers next to the resistance values in the plot indicate the oxygen partial pressure in mbar, at which the respective electrodes were measured.

Electrode surface resistances measured by the i-PLD technique in this study were compared with resistance values from other thin film studies in the literature (including earlier data from our group). As shown in Fig. 3, resistance values found in the literature are usually orders of magnitude higher for all materials. Please note that the data are grouped into low and high *p*O_2_ (values given in the table). All surface resistance values taken from the literature were conducted in conventional *ex situ* measurement setups. From this, we can conclude that standard *ex situ* experiments cannot access the full catalytic capabilities of the studied electrodes. Rather, already degraded states are measured. More specifically, for LSC at both 0.04 mbar and 1000 mbar O_2_, literature data usually report at least 10 times higher surface-related polarization resistances than the values obtained by i-PLD in this study. For LSF, the observed resistance difference is even more pronounced, where different studies report more than two orders of magnitude higher polarization resistances. The same behavior was also observed on STF and PCO electrodes: compared to the literature, up to two orders of magnitude lower surface resistances were found during i-PLD measurements. The occurrence of this trend for a wide range of different materials underlines the general validity of this effect for a broad range of SOFC cathode materials.

### 3.3 Monitoring electrode degradation by simultaneous AP-XPS and *in situ* impedance measurements

In general, the oxygen reduction is known to take place on the surface of the mixed conducting electrodes.^51^ Hence, changes of the electrode surface chemistry can be expected to increase or decrease the activity of the surface towards oxygen reduction. The very low surface-related electrode polarization resistances observed by i-PLD measurements might thus be explained by a virtually uncontaminated and consequently highly active surface of the investigated electrode material. To test this hypothesis, ambient pressure X-ray photoelectron spectroscopy (AP-XPS) measurements were conducted in combination with simultaneous *in situ* electrochemical impedance measurements at different temperatures and oxygen partial pressures. Thus, changes in the surface composition can be correlated with changes of the surface resistance.

For this experiment, an LSC electrode was first prepared and measured by i-PLD, where an area specific resistance of 18 Ω cm^2^ at 600 °C and 0.005 mbar O_2_ was measured. Subsequently, the sample was transferred to the AP-XPS chamber, where XPS measurements at room temperature and UHV conditions found a clean electrode surface with no other contaminants other than adventitious carbon (which is a well-known contaminant on surfaces stored in lab-air and is removed upon heating to 400 °C under AP-XPS conditions) – in particular, no silicon or sulfur was observed.

Besides determination of the overall electrode surface composition, special focus was laid on the detection of sulfur traces on the electrode surface, as sulfur contaminants (typically from SO_2_ and H_2_S in the gas phase) are well known for the poisoning of SOFC cathodes.^11,22,29–43^ To determine the sulfur concentration on the surface of the LSC electrodes, XPS spectra of the S 2p core levels and O 1s core levels were recorded.

**Fig. 4:**
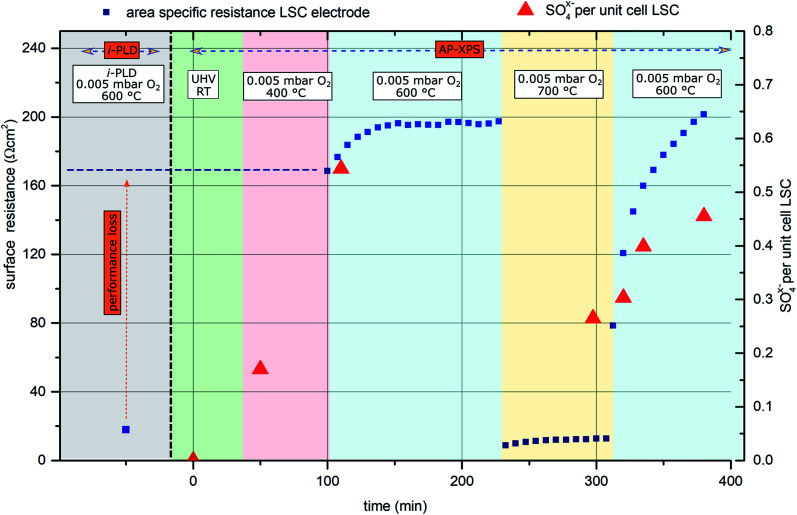
Surface related polarization resistance of an LSC electrode (blue rectangles) measured first by i-PLD and subsequently during AP-XPS. Red triangles represent sulfur oxide species detected on the surface of the electrode (in SO_4_^*x*−^ per (surface) unit cell of the perovskite).

Upon heating the electrode to 400 °C in 0.005 mbar O_2_, XPS measurements found significant amounts of oxidized sulfur on the surface of the sample. This can only be explained by trace impurities of sulfur compounds in the 99.999% high purity oxygen feed gas. Subsequently, the sample was heated to 600 °C, where simultaneous impedance and XPS measurements were carried out. The observed surface resistance (168 Ω cm^2^) was already much higher than the value found in the i-PLD (18 Ω cm^2^), which further degraded for longer annealing times, saturating at 195 Ω cm^2^. The evolution of the surface-related resistance and of the sulfur adsorbate concentration is shown in Fig. 4.

**Fig. 5:**
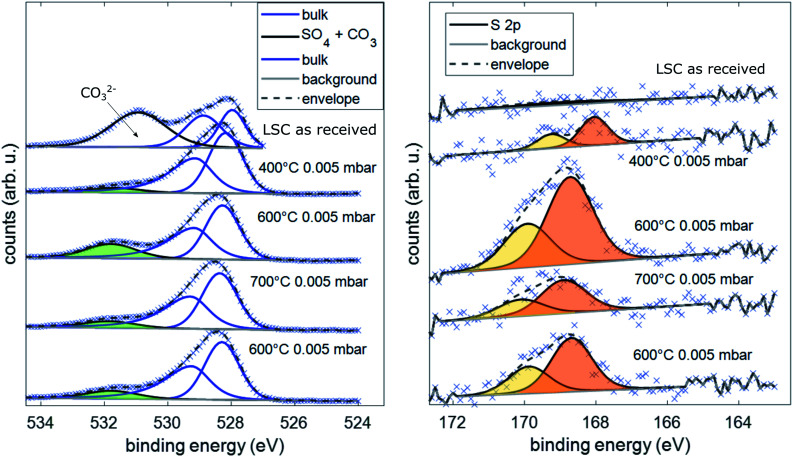
Spectra of the binding energy area of O 1s and S 2p under different conditions during AP-XPS measurements.

Simultaneous to the electrochemical degradation process, the S 2p species at a binding energy of 169–170 eV increased together with an O 1s species at *ca.* 532 eV (see Fig. 5). After correction for photoemission cross-sections and analyzer transmission, a linear relationship of S 2p and 532 eV O 1s atomic fractions with a slope of 0.23 was found (see ESI, Fig. S1†), being in excellent agreement with the stoichiometry of a SO_4_^*x*−^ surface species. The calculated SO_4_^*x*−^ amount per unit cell is plotted in Fig. 4 (0.55 SO_4_^*x*−^ of the unit cell LSC after heating to 600 °C) and already indicates a relationship to the degradation of the surface resistance. Noticeably, various authors of previous studies (including some of the co-authors of this paper)^57–60^ already observed the 532 eV O 1s component on the surface of LSC and related materials and struggled with the assignment due to the unusually high binding energy, compared to perovskite bulk oxygen or typical hydroxide species. Only recently, a correlation of sulfur impurities and the 532 eV O 1s component was found.^60^ It is therefore likely, that sulfur contamination of the electrode surfaces was unnoticed in many studies, due to the very small photoemission cross-section of the S 2p orbitals and the 1 : 4 molar S : O ratio in SO_4_^*x*−^.

Thereafter, the electrode was heated to 700 °C, where a much lower surface resistance was found (10 Ω cm^2^), which is partly caused by the acceleration of the OER kinetics due to temperature activation. Also, a significant decrease of the SO_4_^*x*−^ surface coverage to 0.25 SO_4_^*x*−^ per unit cell LSC was measured. Accordingly, the SO_4_^*x*−^ species are probably not present as a separate phase (*e.g.*: as SrSO_4_), but rather as adsorbed ions with charge compensation taking place in LSC (more oxidized Co in a near-surface (space charge) layer). After cooling the sample down to 600 °C, a lower surface-related polarization resistance (80 Ω cm^2^) was observed. Within 100 minutes, again a strong increase of the surface resistance to around 200 Ω cm^2^ was observed, and simultaneously also the surface coverage with SO_4_^*x*−^ increased again to 0.45 SO_4_^*x*−^ per unit cell LSC. Because of this very clear correlation between SO_4_^*x*−^ coverage and electrochemical electrode activity, it appears very reasonable to directly relate the adsorption and subsequent oxidation of sulfur species such as SO_2_ with the increase of the polarization resistance and hence with electrode deactivation. As already mentioned above, the detrimental effect of SO_2_ on SOFC cathode performance was also observed in previous work.^22,29,61,62^ However, in most studies, SO_2_ was intentionally added to the feed gas in a concentration of a few ppm, and long term degradation effects were investigated.

Moreover, mostly, formation of a SrSO_4_ second phase was either observed or assumed, and degradation was attributed to this second phase. At first glance, it might look surprising that even trace amounts of sulfur contained in high purity measurement gases can poison the surface reaction so quickly. However, assuming a sticking coefficient of 1 and sulfur being present in the form of SO_2_, a dose of 1 langmuir (=1.33 × 10^−6^ mbar × 1 s) is sufficient to cover the surface. Directly after cooling from 700 °C to 600 °C, the SO_4_^*x*−^ coverage increases by *ca.* 1 mono-layer/10 000 s as can be roughly estimated from Fig. 4. Therefore, the SO_2_ partial pressure in the AP-XPS atmosphere seems to be of the order of 10^−10^ mbar. At a total gas pressure of 0.005 mbar, this corresponds to an SO_2_ concentration of at least 0.2 ppm_vol_ in the feed gas, which is in good agreement with impurity analysis of the bottled gas cylinders (see below).

### 3.4 *Ex situ* investigation of pristine surfaces and comparison with the i-PLD results

The AP-XPS measurements showed that already at very low gas phase pressures, trace impurities of sulfur species in high purity gases are sufficient to cause a significant surface coverage with SO_4_^*x*−^ anions. Consequently, further purification of gases is crucial to eliminate all sulfur sources. To address this issue, a gas purification setup was developed to reduce the sulfur contamination in the already high purity (99.999%) measurement gas. A detailed explanation of the setup can be found in the Experimental section.

**Fig. 6:**
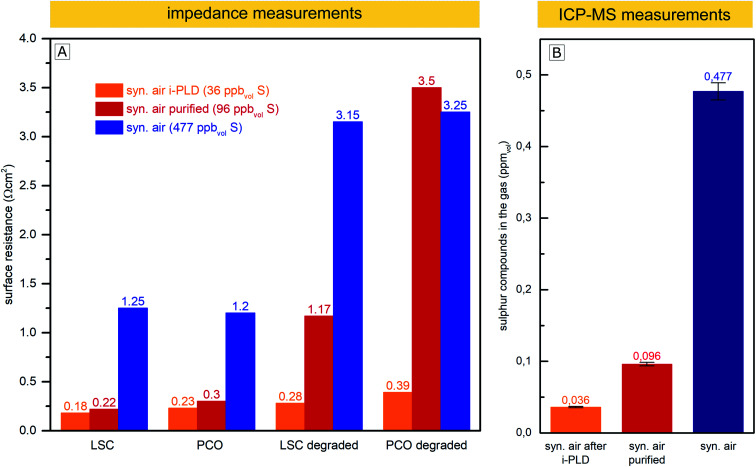
(A) Surface related polarization resistance of LSC and PCO electrodes measured in the i-PLD in synthetic air, in an *ex situ* measurement setup in synthetic air, and *ex situ* with purified synthetic air. Values are shown for pristine electrodes and the same electrodes annealed for 10 h at 600 °C in the corresponding gas (“degraded”). (B) Sulfur compounds found in the measurement feed gas by ICP-MS measurements without purification, after purification with the described setup, and after being passed through the PLD chamber.

In this experiment, the surface-related polarization resistances of LSC and PCO electrodes were measured in three different ways at 600 °C: *in situ* in the i-PLD chamber, *ex situ* in synthetic air (99.999%) in a conventional tubular measurement setup and *ex situ* in the same measurement setup, but with purified synthetic air. The surface resistances of electrodes in a pristine state (directly after heating to 600 °C) and degraded electrodes (annealed for 10 h in synthetic air at 600 °C) were compared and are plotted in Fig. 6. In addition, the feed gas (unpurified, purified with the described setup and after being fed through the PLD) was subjected to ICP-MS measurements to determine the amount of sulfur compounds in the gas. During the sample transfer from the i-PLD to the *ex situ* measurement setup, electrodes were exposed to ambient air at room temperature. However, no contamination with sulfur was observed on the electrode surface after the transfer, as AP-XPS measurements directly after sample transfer have shown (compare Fig. 5).

While 0.477 ppm_vol_ (1.250 μg m^−3^) of sulfur compounds were found by ICP-MS in the feed gas taken from the synthetic air gas bottle provided by the producer (99.999% pure), after gas purification with the described setup, just 0.096 ppm_vol_ (0.251 μg m^−3^) of sulfur compounds remained. We expect the sulfur compounds to be mainly SO_2_ and H_2_S, but with the conducted measurements, we cannot distinguish between different sulfur species. Most interestingly, the sulfur content decreased to an even lower 0.036 ppm_vol_ (0.094 μg m^−3^) after letting the gas flow through the setup of the cold PLD. While this may be surprising at first, note that the inner walls of the PLD chamber (about 0.5 m^2^) are coated with amorphous and highly porous layers of residues from PLD depositions during the 15 years of operation. This “coating”, built up over the years, seems to act as a very efficient getter for sulfur compounds and most probably also for other contaminants, thus explaining why electrodes during i-PLD measurements experience such low degradation rates. For an in-depth characterization of the “coating”, X-ray fluorescence spectroscopy (XRF) measurements were conducted, which revealed the PLD walls to be mainly composed of La (20.4%), Mn (18.6%), Co (13.2%) and Sr (11.8%)) oxides; please see Fig. S4 in the ESI† for precise composition. A high sticking coefficient is expected for sulfur on these oxides.^63^ Moreover, also considering the area difference between the PLD wall (0.5 m^2^) and the area of the investigated thin film electrode (0.5 cm^2^), absorption of SO_2_ is far more likely on the PLD walls.

As can be seen in Fig. 6, a clear correlation can be found between the concentration of sulfur compounds detected in the feed gas and the measured pristine electrode surface resistance. In the i-PLD, electrodes showed very low resistances, and also upon annealing, only a slight increase of the surface resistance was observed. The surface composition of LSC and PCO electrodes was investigated by XPS measurements after annealing for 12 h in synthetic air in the setup of the i-PLD, and as expected, no sulfur contamination was found on the electrode surface, (see ESI S1†).

*Ex situ* measurements with the feed gas from unpurified synthetic air (99.999%) yielded significantly higher surface-related polarization resistances with electrodes having around an order of magnitude larger resistances compared to the values found during i-PLD measurements, being in good agreement with the usual difference between *in situ* and *ex situ* measurements performed in our group. Measurements with synthetic air purified using the described setup, where a large extent of sulfur was removed from the feed gas, showed very low surface resistances compared to unpurified synthetic air, almost reaching the extremely low values found during i-PLD measurements. The slightly higher surface resistance found during impedance measurements with purified synthetic air compared to measurements in the i-PLD may already reflect the sulfur concentration of both atmospheres, thus highlighting the high sensitivity of the electrode surfaces towards sulfur poisoning. It is further worth mentioning that the sudden strong performance decrease during *ex situ* measurements was observed with different gas composition/mixtures (premixed synthetic air (5.0) as well as synthetic air mixed in our laboratory with MFCs from oxygen 5.0 and nitrogen 5.0) and was also independent from the gas bottle provider (Messer™ or Airliquide™). With regard to the long-term stability of the surface-related polarization resistance, the measurements revealed very little degradation inside the PLD chamber, while during *ex situ* measurements, a much stronger increase of the resistance values was observed, despite additional purification. This might be caused by residual contaminants from the measurement setup (*e.g.* steel or quartz parts of the setup). This assumption is supported by the fact that gas purification only showed a significant effect if the *ex situ* measurement setup was baked out at 800 °C before the measurement, thus removing residual contaminants. Moreover, the coated i-PLD heater may also act as a sulfur getter and further decrease the amount of sulfur on the sample surface.

## 4 Discussion

In this chapter, we summarize our findings regarding the high activity of pristine, contamination-free MIEC cathodes and their degradation, suggest mechanistic explanations and discuss them in the context of recent findings in the literature. Overall, the following experimental facts have to be considered:

(i) All investigated SOFC cathode materials (LSF, LSC, STF, PCO and Pt doped LSF) measured in the i-PLD setup exhibit very low surface resistances both at 0.04 mbar and 1000 mbar O_2_.

(ii) Surface-related polarization resistances reported in the literature are usually one or even two orders of magnitude higher than the resistances found during i-PLD measurements.

(iii) *In situ* AP-XPS measurements revealed a clear correlation between the presence of an SO_4_^*x*−^ species on the electrode surface and a strong performance loss of the investigated LSC electrode.

(iv) The surface coverage of these species and ease of desorption at 700 °C strongly suggest that a separate SrSO_4_ phase has not formed yet.

(v) Sulfur contaminants were detected in the high purity measurement gas (99.999%), and their removal using a gas purification setup allowed to achieve significantly lower surface-related polarization resistances.

Based on these results, we have strong reason to believe that large parts of the performance degradation of pristine, super clean SOFC electrodes are directly correlated to minor sulfur contamination. As we have shown, trace amounts of sulfur in the feed gas from high purity (99.999%) gas bottles – even when they are used in an AP-XPS setup with SO_2_ partial pressures < 10^−9^ bar – already cause a saturation of the surface with SO_4_^*x*−^ species and a significant increase of the surface polarization resistance. Thus, we suggest that in virtually all studies conducted on thin film electrodes reported in the literature, sulfur contamination was also present, albeit mostly unperceived.

In general, the effect of sulfur poisoning of SOFC cathodes has already been intensely studied in the literature on cathode materials such as LSM,^30–33^ LSC,^22,34–36^ BSCF,^37^ or LSCF,^38–42^ and the fact that sulfur contaminants are detrimental for the surface exchange kinetics is not surprising. In addition, the formation of a SrSO_4_ secondary phase was frequently observed.^22,29,31–34,36,37,64^ However, most studies were conducted with higher *p*(SO_2_), which was intentionally added and/or at higher temperatures and/or for longer degradation times. For example, Bucher *et al.* reported that trace impurities of a few ppb SO_2_ present in nominally pure measurement gases are responsible for a performance loss of LSC electrodes^22^ by a factor of 10, but this degradation took place over a time period of 1000 h. In contrast, we found a nearly instantaneous dramatic performance loss, which is presumably omnipresent, but went unnoticed in most experiments so far. Accordingly, we are confident that the sulfur poisoning considered here (*i.e.* the formation of a charged adsorbate layer) is fundamentally different from sulfur poisoning due to a second phase formation as discussed in ref. 22, 29, 31–34, 36, 37 and 64, see also above.

**Fig. 7:**
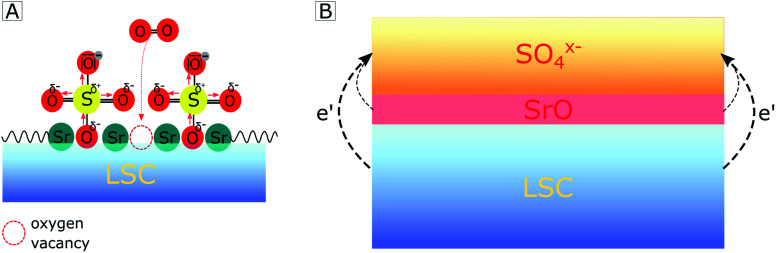
Sketch showing how adsorption of SO_4_^*x*−^ on a LSC electrode surface hampers oxygen adsorption by (A) steric effects and (B) by a charge transfer from the electrode subsurface region to sulfate adsorbates, thus forming a space charge beneath.

With regard to the detailed mechanism of how SO_2_ reversibly deactivates the electrode surface, it is important to note that the oxygen reduction mechanism itself is not fully understood yet. However, we will suggest a realistic hypothesis for the deactivation process, which explains our (and a variety of other) experimental results. We assume that SO_2_ adsorbs on the electrode surface by forming a bond at an O lattice site and that it is subsequently oxidized to SO_4_^*x*−^ by directly or indirectly involving gaseous oxygen. At this point, it is noteworthy that the final equilibrium is independent of whether SO_2_ adsorbs directly on an O lattice site or in a surface vacancy, as previously proposed by Wang *et al.* on LSCF.^42^ In accordance with an increase of the work function^65^ and differences in electronegativity,^66^ we propose that the SO_4_^*x*−^ adsorbate layer extracts the charge of the electrode material below which consequently leads to a negatively charged surface and to the formation of a subsurface space charge (see Fig. 7B). A quite similar formation of a subsurface space charge can be observed on Taguchi sensors,^67^ where the charge is transferred from an SnO_2_ surface to adsorbed O_2_ molecules.

The consequences of this altered sulfate-rich surface on the oxygen exchange kinetics are manifold. First, already due to steric reasons, *i.e.* by blocking adsorption sites (see Fig. 7A), the oxygen exchange rate is decreased. Second, electronic charge carriers enter the rate equation of the oxygen reduction reaction, and a reduced electron concentration (or an increased electron hole concentration) is expected to lower the ORR rate and thus increases the electrode polarization resistance. Third, the sulfate adsorbates accumulate negative charges and reduce the probability of charge transfer to adsorbed oxygen molecules, which is an essential step in the path of the oxygen reduction reaction. It is worth mentioning that even without any additional charge, the bulky adsorbates on top of the electrode may cause a substantial surface potential (*χ*) increase due to a dipole-like charge redistribution.

In addition to explaining our experimental results, this hypothesis is also in accordance with a recent study by Nicollet *et al.* on PCO,^68^ where a strong correlation between the oxygen exchange kinetics and the relative acidity^69^ (which is the ionic equivalent of the here-proposed work function/electronegativity difference) of decorated surface oxides was found (acidic oxides are *e.g.* SiO_2_, CrO_3_ or SO_2_ and SO_3_, while basic oxides are *e.g.* CaO, Li_2_O or SrO). Moreover, we may link the model presented here (degradation by SO_4_^*x*−^ adsorbates) to reports on Sr segregation as the main reason for the degradation of Sr-containing electrodes.^21,23–28^ In all these studies, no specific removal of trace amounts of sulfur compounds from the feed gases was performed, and presumably, electrode surfaces were therefore *a priori* poisoned by SO_4_^*x*−^ species. Several studies by us and by other groups likely report signs of sulfur contamination on the electrode surface in the O 1s binding energy region (532 eV) of XPS spectra, which were not discussed or possibly wrongly assigned to other chemical compounds. For all these studies, additional XPS measurements in the binding energy region of sulfur were not conducted. Therefore, we suggest that the surface dipole layer may significantly accelerate Sr segregation to the electrode surface, and the here-suggested SO_4_^*x*−^ adsorption is simply the first step of subsequent SrSO_4_ formation with ongoing Sr segregation, which causes further long-term degradation. This is supported by several studies in the literature which found other contaminants such as Cr,^70,71^ H_2_O^71–74^ and CO_2_ (ref. 75) to accelerate Sr segregation. Thus, it would be interesting to study Sr segregation in highly pure, sulfur-free measurement feed gases and compare those with the current results.

## 5 Conclusion

Impedance measurements on thin film electrodes within the PLD chamber (i-PLD) revealed that the pristine surfaces of several promising cathode materials for intermediate temperature solid oxide fuel cells (LSC, LSF, PCO, and STF) possess by far better oxygen reduction kinetics than determined in previous studies using common electrochemical *ex situ* characterization. The surface related polarization resistances measured during i-PLD were often one to two orders of magnitude lower than values previously determined in the literature. In order to clarify the origin of this huge difference, ambient pressure XPS measurements with simultaneous impedance measurements were carried out after i-PLD impedance measurements. These experiments revealed the formation of SO_4_^*x*−^ species on the electrode surface, even when using high purity gases at very low pressures. It could be shown, that these SO_4_^*x*−^ adsorbates clearly correlate with severe degradation of the oxygen reduction kinetics. A gas purification setup was used to further clean the high purity feed gases, and the corresponding removal of sulfur compounds from the feed gas led to very low surface polarization resistances in conventional *ex situ* measurement setups which were close to i-PLD values. While sulfur is well known for its detrimental effect on the oxygen exchange kinetics of fuel cell cathodes during long-time degradation experiments, this study shows that the very high inherent activity of these materials is already substantially reduced by trace amounts of sulfur in nominally pure feed gases. The corresponding initial very quick but still reversible degradation step has to be separated from any subsequent degradation by SrSO_4_ second phase formation, even though it might be an important driving force for the latter or for the well-known Sr segregation. This virtually omnipresent (but significant) degradation often goes unnoticed, but is crucial for the development of novel SOFC materials, as it further emphasizes the importance of the sulfur resilience of cathode surfaces.

## Conflicts of interest

There are no conflicts to declare.

## Supplementary Material

TA-010-D2TA03335F-s001
