# Supplementary material for: In situ techniques reveal the true capabilities of SOFC cathode materials and their sudden degradation due to omnipresent sulfur trace impurities
Source: J Mater Chem A Mater. 2022 Jun 23;10(28):14838–48. doi: 10.1039/d2ta03335f (PMC9295724; doi:10.1039/d2ta03335f)
Supplement: TA-010-D2TA03335F-s001 [file TA-010-D2TA03335F-s001.pdf]

Electronic Supplementary Material (ESI) for Journal of Materials Chemistry A.
This journal is © The Royal Society of Chemistry 2022

Supporting Information: *In-situ* techniques reveal the true capabilities of SOFC cathode materials and their sudden degradation due to omnipresent sulfur trace impurities

Christoph Riedl,‡[a] Matthäus Siebenhofer,*[a,c] Andreas Nenning,[a] Alexander Schmid,[a] Maximilian Weiss,[a] Christoph Rameshan,[b] Andreas Limbeck,[a] Markus Kubicek,[a] Alexander Karl Opitz,[a] Juergen Fleig[a]*

[a] Institute of Chemical Technologies and Analytics

TU Wien

Getreidemarkt 9-E164, 1060 Vienna, Austria

‡ E-mail: christoph.riedl@tuwien.ac.at

[b] Institute of Materials Chemistry

TU Wien

Getreidemarkt 9-E165-PC, 1060 Vienna, Austria

[c] CEST Kompetenzzentrum für elektrochemische Oberflächentechnologie GmbH

TFZ – Wiener Neustadt Viktor-Kaplan-Strasse 2

2700 Wiener Neustadt, Austria

*Authors contributed equally.

1 XPS spectra of LSC electrode during AP-XPS measurements:

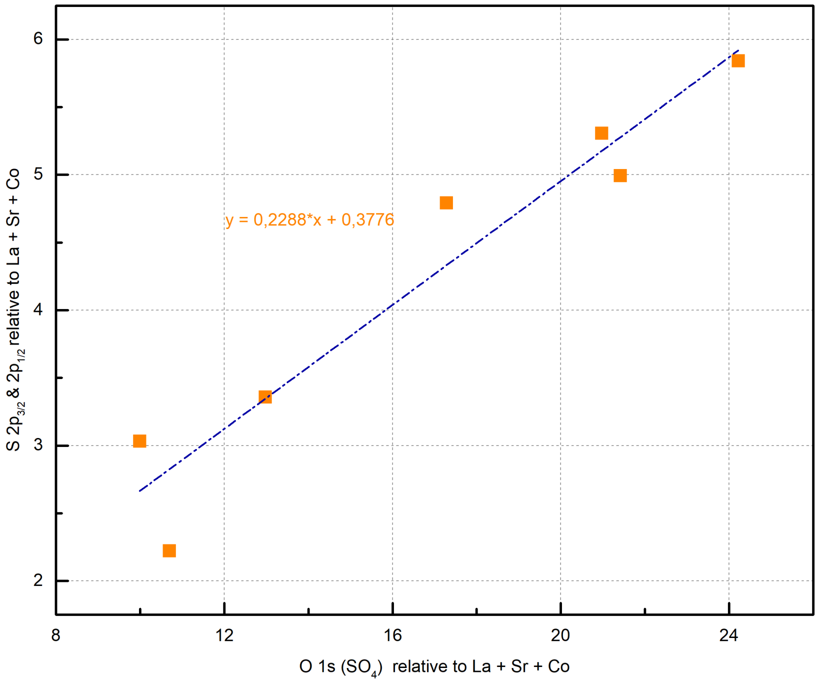

Figure S1: Linear correlation between the S 2p peak area (relative to La + Sr + Co) plotted versus the O 1s (SO_4) area (relative to La + Sr + Co). The observed slope corresponds well with the stoichiometric ratio of SO_4

.

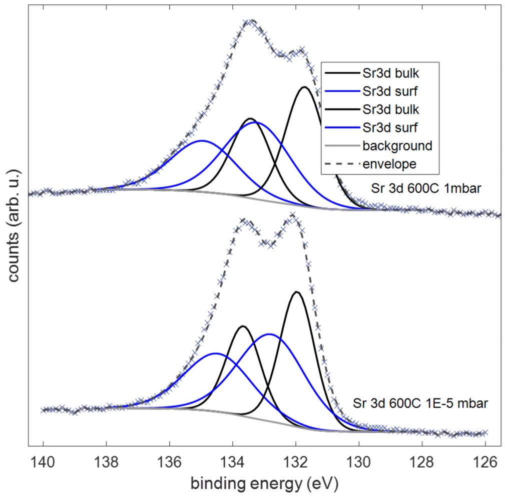

Figure S2: Spectra in the Sr 3d binding energy region of the LSC electrode.

In Figure S1 the S 2p peak area is plotted versus the O 1s area. Linear fitting of the data points yields a slope of 0.228, which corresponds well to the stoichiometric ratio observed in SO_4. More-

over, in Figure S2 the Sr 3d binding energy region is plotted. The measurements revealed that no significant change in the Sr 3d binding energy region occurred over time, which allows us to exclude Sr segregation as reason for the strong performance loss observed in our measurements. The Sr-3d spectra recorded at 10^{-5} mbar were measured on a pristine SO_4 free surface, whereas a significant SO_4 coverage was detected when conducting the measurements at 1 mbar. Mostly likely the counter ion for SO_4 is a surface strontium ion. Therefore the surface strontium ions undergo a chemical transition from SrO-like to $SrSO_4$-like surface terminations, which is reflected by a change in the binding energy of the Sr-3d component.

2 LSC and PCO electrodes directly after *i*-PLD measurements:

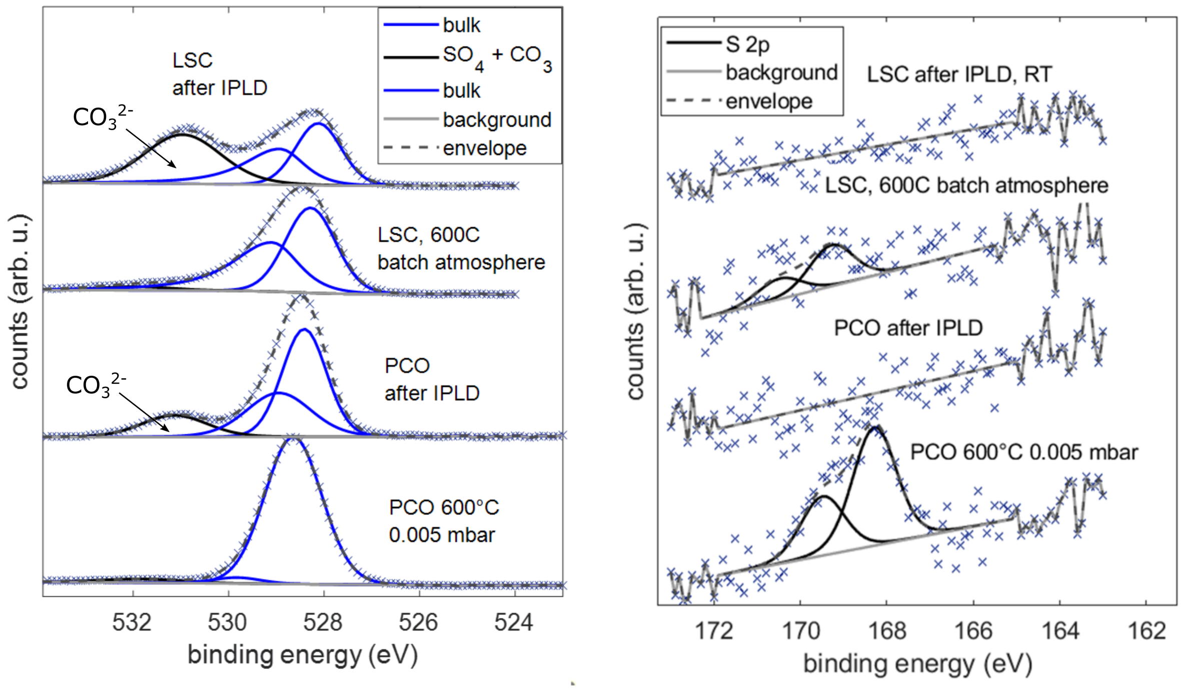

Figure S3: Spectra of O 1s and S 2p binding energy of an LSC and PCO electrode directly after *i*-PLD measurements.

The investigated LSC and PCO electrodes were annealed at 600 °C in synthetic air in the *i*-PLD for 12 h. As visible in figure S3, conducted XPS measurements revealed sulfur free surfaces directly after the *i*-PLD. After heat up of the eletrodes to 600 °C in the setup of the AP-XPS, sulfur contaminations were detected on the electrode surface. This is in excellent accordance to the superior oxygen exchange kinetics observed during *i*-PLD measurements.

3 X-ray fluorescence spectroscopy (XRF):

In Figure S4 the powder obtained from the PLD walls was analyzed by X-ray fluorescence spectroscopy (XRF). XRF found that the powder was mostly composed of La (20,4 %), Mn (18,6 %), Co (13,2 %) and Sr (18,6 %). It was not possible to detect residual amounts of sulfur as the emission lines of sulfur overlap with emission lines of Co.

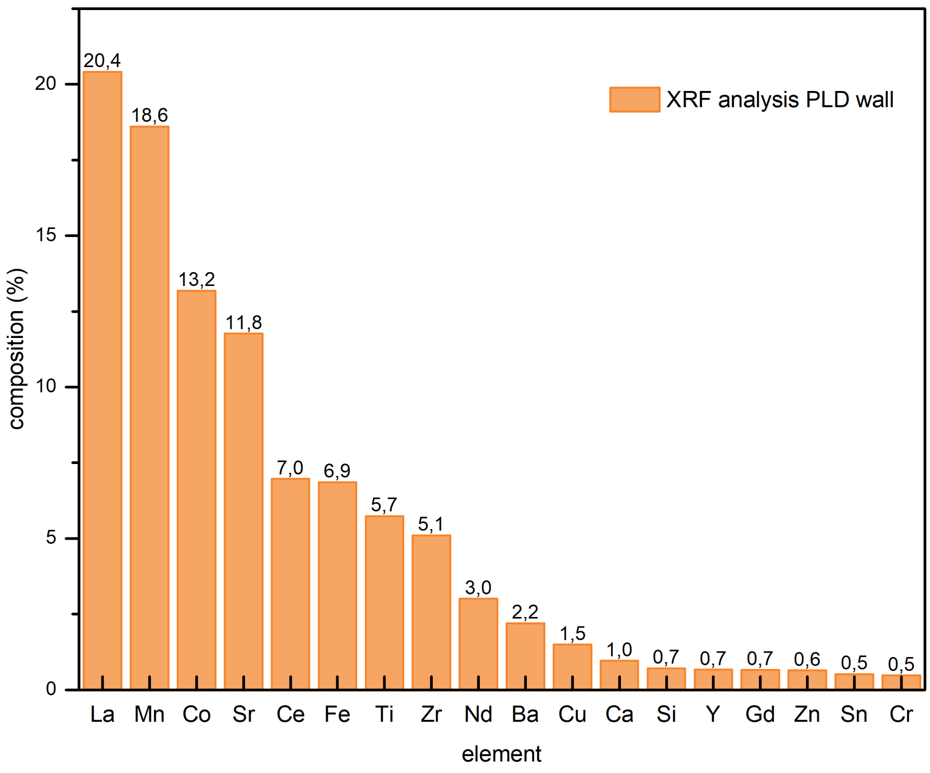

Figure S4: Analysis of powder scraped from the PLD wall.
